# Effects of the C57BL/6 strain background on tauopathy progression in the rTg4510 mouse model

**DOI:** 10.1186/1750-1326-9-8

**Published:** 2014-01-15

**Authors:** Rachel M Bailey, John Howard, Joshua Knight, Naruhiko Sahara, Dennis W Dickson, Jada Lewis

**Affiliations:** 1 Center for Translational Research in Neurodegenerative Disease and Department of Neuroscience, University of Florida, Gainesville, FL 32610, USA; 2 Department of Neuroscience, Mayo Clinic, Jacksonville, FL 32224, USA

**Keywords:** Transgenic mouse models, Strain background, C57BL/6, rTg4510, Tau, Tauopathy, Neurodegeneration, Behavior, Morris water maze

## Abstract

**Background:**

Cross-breeding of transgenic mice is commonly used to assess gene-gene interactions, particularly in the context of disease. Strain background changes can influence the phenotype of mouse models and can confound crossbreeding studies. We sought to determine if changing the strain background of a commonly used mouse model of tauopathy (rTg4510) would significantly impact the originally reported phenotype. On the original F1 FVB/N x 129S6 background, rTg4510 mice present with progressive cognitive decline, increased insoluble tau, robust tau pathology and age-dependent neurodegeneration. One of the most common strains in mouse modeling is C57BL/6. We and others have previously reported that this strain background alters the phenotypes of various models, including the JNPL3 model of tauopathy. To determine if the phenotype of rTg4510 mice was similarly affected by the introduction of the C57BL/6 background, we compared rTg4510 mice on the original F1 FVB/N x 129S6 background to rTg4510 mice on an F1 FVB/N x C57BL/6NTac (B6/NTac) background, herein termed rTg4510_B6_.

**Results:**

Despite a small, but significant increase in soluble human tau levels, young rTg4510_B6_ mice had equivalent levels of tau phosphorylation, aggregation and cognitive impairments as age-matched rTg4510 mice. At 6.5 months of age, rTg4510_B6_ mice displayed hyperphosphorylated insoluble tau and robust cortical tau neuropathology that was equivalent to age-matched rTg4510 mice; however, 10.5-month-old rTg4510_B6_ mice had greater amounts of phospho-tau in the cortex and hippocampus when compared to age-matched rTg4510 mice. Non-transgenic (NT) littermates of rTg4510_B6_ (NT_B6_) mice also had greater amounts of cortical and hippocampal phospho-tau at 10.5 months of age when compared to NT littermates of rTg4510 mice. Additionally, older rTg4510_B6_ mice had gross forebrain neurodegeneration that was equivalent to age-matched rTg4510 mice.

**Conclusions:**

Overall, our data shows that introduction of the C57BL/6 strain into the rTg4510 mouse background modestly alters the tau pathology that was originally reported in rTg4510 on the F1 FVB/129 background. In contrast, behavioral and neurodegenerative outcomes were not altered. These studies support the use of the rTg4510 mouse model on a partial C57BL/6 strain background without losing fidelity of the phenotype and suggest that the C57BL/6 background does not inherently protect against tauopathy.

## Background

Numerous transgenic mice expressing wild-type or mutant human tau have been created to model the neuropathology of tauopathies–a group of neurodegenerative diseases characterized by the accumulation of tau protein aggregates. Many of the behaviors and pathologies reported for these mouse models, even those that express the same tau protein, are different. Several factors may contribute to these differences, including the levels and regions of transgenic tau protein expression, the characteristics of the promoters used to drive transgenic tau expression, and the behavioral tests used to characterize the models. The strain background of transgenic mice can also alter disease progression and presentation.

**Figure 1:**
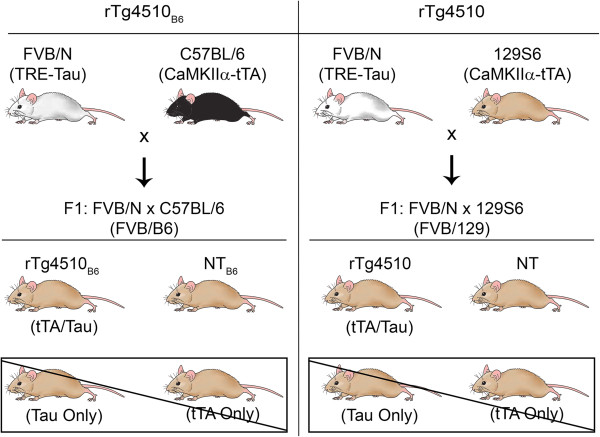
**Breeding scheme for comparison of rTg4510 mice on different strain backgrounds.** Breeding scheme used to produce rTg4510 mice on an F1 FVB/N x C57BL/6 (FVB/B6) strain background (rTg4510_B6_) (Left) compared to the original rT4510 mice on an F1 FVB/N x 129S6 (FVB/129) strain background (Right). Mice used in these studies include rTg4510_B6_ and rTg4510 mice that carry both the tau responder and the tTA activator transgenes and non-transgenic (NT_B6_ and NT) mice. Single transgenic mice generated from these F1 crosses were not analyzed in these studies.

JNPL3 [1] and rTg4510 [2] mice are two widely used, independent mouse models of human tauopathy that express the same human 0N4R tau protein using different promoter systems. Previously, Bolmont et al. crossed the JNPL3 mouse model from a mixed background onto an inbred C57BL/6 J background and found that the regional distribution of tauopathy was altered and that the timing of phenotype was significantly delayed [3]. Studies have also suggested that the C57BL/6 background is protective against neurotoxicity in other disease models [4-7]. To test the hypothesis that crossing rTg4510 mice to a C57BL/6 strain background is protective against tauopathy, we have compared rTg4510 mice on the original F1 FVB/N x 129S6 background to rTg4510 on an F1 FVB/N x C57BL/6NTac background (rTg4510_B6_) (Figure 1). Here we compare the phenotypes of both young and old rTg4510 mice on each strain background.

## Results

### Increased human tau levels in young rTg4510_B6_ mice

**Figure 2:**
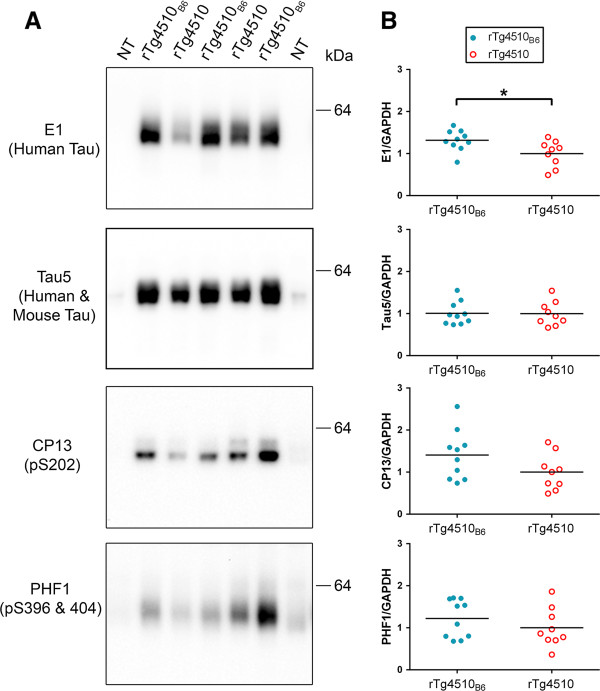
**2.5-month-old rTg4510**_**B6 **_**mice have increased human tau expression compared to rTg4510 mice. (A-B)** Western blot analyses of the soluble fractions of whole brain lysates from rTg4510_B6_ and rTg4510 mice at 2.5 months of age. **(A)** Representative western blots of soluble human tau (E1 antibody), human and mouse tau (Tau5 antibody), and phosphorylated tau (CP13 and PHF1 antibodies) with a NT littermate shown as a negative control. **(B)** Densitometric quantification of E1 (human tau), Tau5 (human and mouse tau), CP13 (pS202 tau) and PHF1 (pS396/404 tau) normalized to GAPDH. rTg4510_B6_ mice had increased soluble human tau, indicating greater tau transgene expression than age-matched rTg4510 mice. No differences in soluble phospho-tau levels were detected using CP13 and PHF1 antibodies. Each dot represents an individual mouse with the mean indicated by the black line. n = 9-10 per cohort. *P < 0.05 (Student’s t-test).

Tau is normally a highly soluble protein and expression levels are generally measured in soluble protein extracts. In order to compare human tau expression levels in rTg4510_B6_ and rTg4510 mice, we prepared soluble brain extracts from mice at 2.5 months of age, prior to robust tau pathology and neuronal loss. rTg4510_B6_ mice had a small, but significant increase (0.32 ± 0.13; p < 0.05) in soluble human tau levels compared to rTg4510 mice at 2.5 months of age, suggesting that rTg4510_B6_ mice may have moderately elevated tau transgene expression (Figure 2). Using the Tau5 antibody that recognizes both the transgenic human tau and endogenous mouse tau, no differences in soluble total tau levels were found between rTg4510_B6_ and rTg4510 mice at 2.5 months of age. Human tau expression in the rTg4510 lines is controlled by the tetracycline transactivator (tTA). Although both the tTA_129_ mice used for rTg4510 mice and the tTA_B6_ mice used for rTg4510_B6_ mice originated from the same tTA line [8], this minor increase in tau expression could be due to minor changes in tTA expression on the two backgrounds. We were, however, unable to assess tTA expression due to the lack of a good tTA antibody, a problem that is consistent with data from others working with tTA mouse lines [5,9,10].

### Tau was biochemically equivalent between young rTg4510_B6_ and young rTg4510 mice

Normal tau function and solubility is regulated by phosphorylation (reviewed in [11]); therefore, we compared the phosphorylation of soluble tau in young rTg4510 mice on both strain backgrounds. To detect tau phosphorylated at S202 and S396/404–sites that are hyper-phosphorylated in human tauopathies–we used the phospho-tau antibodies CP13 and PHF1, respectively [2,12-14]. Analysis of soluble brain extract with the CP13 and PHF1 antibodies revealed that young rTg4510_B6_ mice had similar phosphorylation of soluble tau as rTg4510 mice (Figure 2).

**Figure 3:**
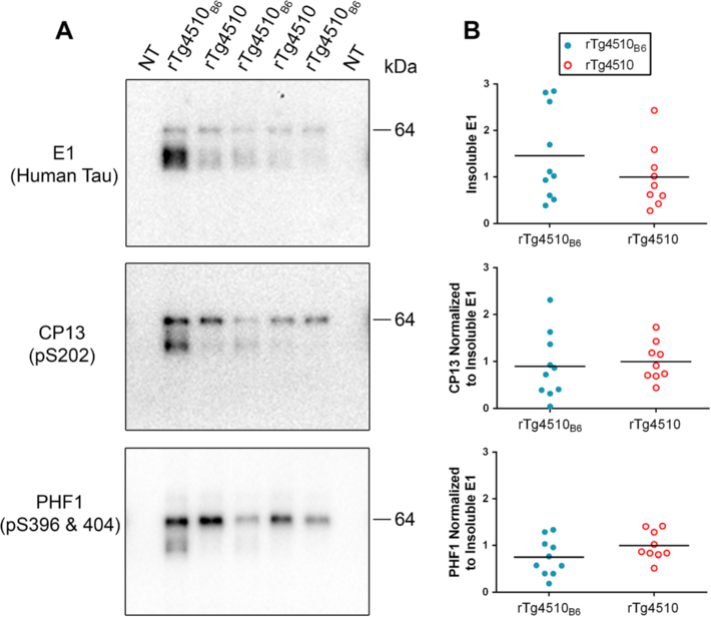
**Equivalent accumulation and phosphorylation of insoluble tau in 2.5-month-old rTg4510**_**B6 **_**and rTg4510 mice. (A-B)** Western blot analyses of the sarkosyl-insoluble fractions of whole brain lysates from rTg4510_B6_ and rTg4510 mice at 2.5 months of age. **(A)** Representative western blots of insoluble human tau (E1 antibody) and phosphorylated tau (CP13 and PHF1 antibodies) with a NT littermate shown as a negative control. **(B)** Densitometric quantification of E1 (human tau), CP13 (pS202 tau) and PHF1 (pS396/404 tau). Phospho-tau is normalized to the amount of human tau aggregated in that fraction. Insoluble tau accumulation and phosphorylation were similar between rTg4510_B6_ and rTg4510 mice. Each dot represents an individual mouse with the mean indicated by the black line. n = 9-10 per cohort.

A central feature of human tauopathies is the loss of tau solubility and the accumulation of hyper-phosphorylated tau in aggregates that can be isolated from brain with sarkosyl extraction [15]. Tau deposition in the sarkosyl-insoluble brain fraction can be detected as early as 2.5 months of age in rTg4510 mice. With increasing age and disease progression, insoluble tau shifts from a ~55 kDa species to a hyper-phosphorylated 64 kDa species [14] and we have previously demonstrated that 64 kDa tau is biochemically equivalent to NFTs in tau transgenic mice [1]. At 2.5 months of age, there was no significant difference in the accumulation of insoluble human tau and the phosphorylation of insoluble tau in rTg4510_B6_ mice compared to rTg4510 mice (Figure 3). Importantly, we observed the initial aggregation of a 64 kDa tau species in rTg4510 mice on either strain background. There was considerable spread in the degree of insoluble tau found within each group, consistent with each animal being in slightly different stages of the initial, rapid development of tauopathy. We also examined the insoluble fraction with the Tau5 antibody, but were unable to obtain sufficient Tau5 antibody reactivity with the insoluble fraction from 2.5 month-old rTg4510 mice on either strain background (data not shown).

### Comparable cognitive deficits in young rTg4510_B6_ and rTg4510 mice

**Figure 4:**
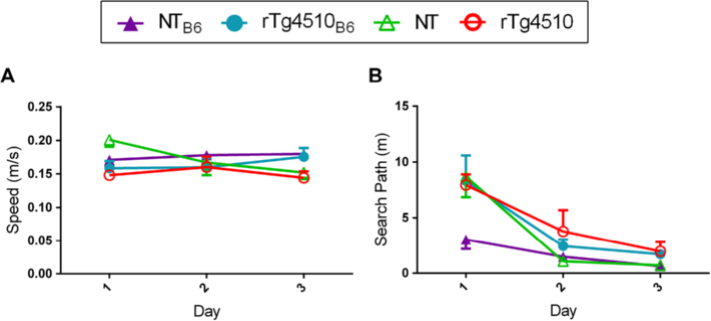
**Strain background does not alter swim speed or search path in the MWM. (A-B)** Performance in the cued MWM task was equivalent amongst rTg4510 and NT littermates on either strain background at 2.5 months of age. **(A)** Swim speeds to the visible platform were equivalent across all groups. **(B)** All groups improved performance over training (p < 0.001) with the search paths to reach the visible platform longer for rTg4510 mice on days 1 and 2, but comparable to NT mice by day 3. No differences between strain backgrounds were detected. Data expressed as mean ± SEM and analyzed via multifactorial (genotype x strain) RM ANOVA with *post hoc* Bonferonni’s multiple comparisons test. n = 5 per cohort.

The initial characterization of rTg4510 mice showed that human tau expressing mice had similar motor performance to tau negative mice on the F1 FVB/129S strain background at 2.5 months of age, before widespread neurodegeneration in the brain [10]. Studies have shown, however, that non-transgenic (NT) mice on different strain backgrounds can have variable motor performance in a variety of behavioral tasks [16]. To assess effects of the C57BL/6 strain background on sensorimotor function, 2.5-month-old rTg4510_B6_, rTg4510 and NT mice on both strain backgrounds performed the cued (visible platform) version of MWM. No differences in swim speed were observed across training between all groups (Figure 4A). Further, all groups improved performance over training, as indicated by the decreased search path to the platform [F (2, 32) = 35.91, p < 0.001], but rTg4510 mice on both strain backgrounds had longer search paths to reach the platform than NT mice [F (1, 16) = 5.39, p < 0.05] (Figure 4B). *Post hoc* analysis revealed that by the third day of visible platform training, all groups swam comparable distances to reach the platform. Equivalent results were found with measurements of the escape latency to reach the platform (data not shown). Importantly, no differences between strains were detected, signifying that mice on an F1 FVB/B6 background had similar sensorimotor function as mice on the F1 FVB/129 background.

**Figure 5:**
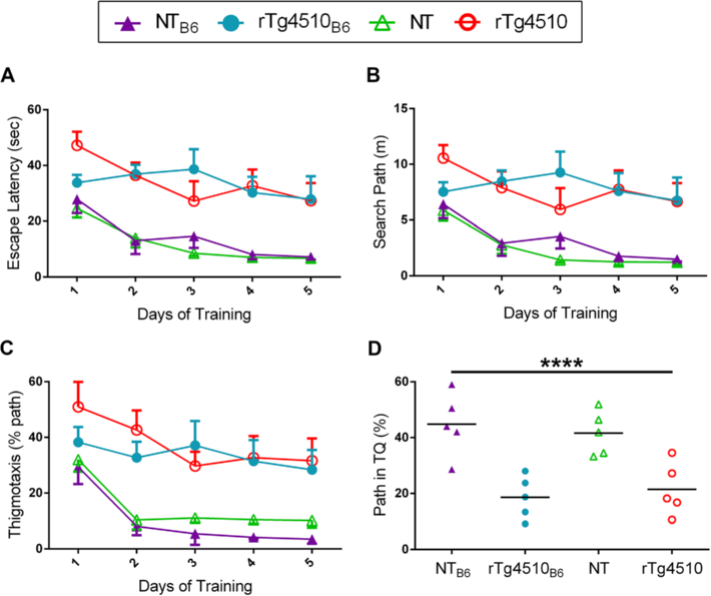
**rTg4510**_**B6 **_**and rTg4510 mice have equivalent cognitive deficits at 2.5 months of age.** Spatial learning and memory was equivalently impaired in rTg4510_B6_ and rTg4510 mice at 2.5 months of age. **(A)** rTg4510 mice took significantly more time to find the hidden platform than NT mice (p < 0.001), with no difference between the strain backgrounds. **(B)** The search paths to reach the hidden platform for both rTg4510_B6_ and rTg4510 mice were equivalent and significantly longer than the search paths of NT mice (p < 0.001). **(C)** rTg4510_B6_ and rTg4510 mice also had similar thigmotaxic swimming that was significantly longer than that of NT mice (p < 0.001). **(D)** Spatial memory was equally impaired in rTg4510_B6_ and rTg4510 mice compared to age-matched NT mice as indicated by the decreased percentage of distance traveled in the target quadrant (TQ) during the final probe trial. **(A-C)** Data expressed as mean ± SEM and analyzed via multifactorial (genotype x strain) RM ANOVA. **(D)** Each dot represents an individual mouse with the mean indicated by the black line. ****p < 0.0001 [two-way ANOVA (genotype x strain): main effect of genotype indicated]. n = 5 per cohort.

Spatial learning and reference memory are hippocampal dependent functions [17]. The hippocampus is one of the first regions affected by tauopathy in rTg4510 mice and cognitive deficits in the hidden platform version of MWM are detected as early as 2.5 months of age in rTg4510 on the original F1 FVB/129 strain background [2,14]. Analysis of our 2.5-month-old rTg4510 and NT cohorts showed improvement in finding the hidden platform for all groups across training days [escape latency: F (4, 64) = 8.43, p < 0.001), search path: F (4, 64) = 5.77, p < 0.001] (Figure 5A-B). rTg4510 mice showed significantly less improvement in performance than NT mice as indicated by longer escape latencies [F (1, 16) = 61.78, p < 0.001)] (Figure 5A) and longer search paths to find the hidden platform [F(1, 16) = 48.83, p < 0.001)] (Figure 5B). Additionally, no strain background effect was detected for either parameter. Irrespective of strain background, rTg4510 mice showed increased thigmotaxic, or wall-hugging, swimming compared to NT mice [F (1, 16) = 24.70, p < 0.001]. The thigomotaxic behavior of rTg4510 mice decreased over training days [F (4, 64) = 19.07, p < 0.001]; however, it never reached the low levels of thigomotaxis observed in NT mice (Figure 5C). Spatial learning performance was also assessed by the percentage of distance swam in the target quadrant during the final probe trial–rTg4510 mice on both strain backgrounds were significantly impaired compared to NT mice (p < 0.0001) (Figure 5D). Overall, we found that crossing rTg4510 mice to a C57BL/6 strain background did not affect sensorimotor function or attenuate the cognitive decline that is characteristic of rTg4510 mice on the original strain background at 2.5 months of age.

### Characterization of tau in older rTg4510_B6_ brain extracts

**Figure 6:**
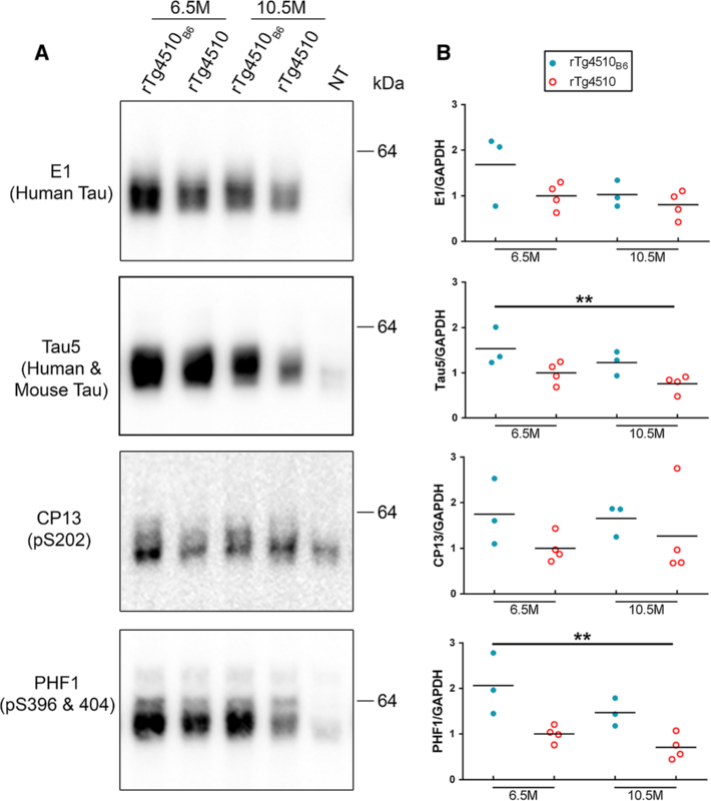
**Older rTg4510**_**B6 **_**mice have increased soluble Tau5 and PHF1 levels compared to rTg4510 mice. (A-B)** Western blot analyses of the soluble fractions of whole brain lysates from 6.5- and 10.5-month-old rTg4510_B6_ and rTg4510 mice. **(A)** Representative western blots of soluble human tau, human and mouse tau, and phosphorylated tau and with a NT littermate shown as a negative control. **(B)** Densitometric quantification of E1 (human tau), Tau5 (human and mouse tau), CP13 (pS202 tau) and PHF1 (pS396/404 tau) normalized to GAPDH. rTg4510_B6_ mice had equivalent levels of soluble CP13 and human tau, and increased soluble total tau (human and moues) and PHF1 tau compared to rTg4510 mice on the original strain background. Each dot represents an individual mouse with the mean indicated by the black line. n = 3-4 per cohort. **p < 0.01 [two-way ANOVA (strain x age): main effect of strain indicated].

**Figure 7:**
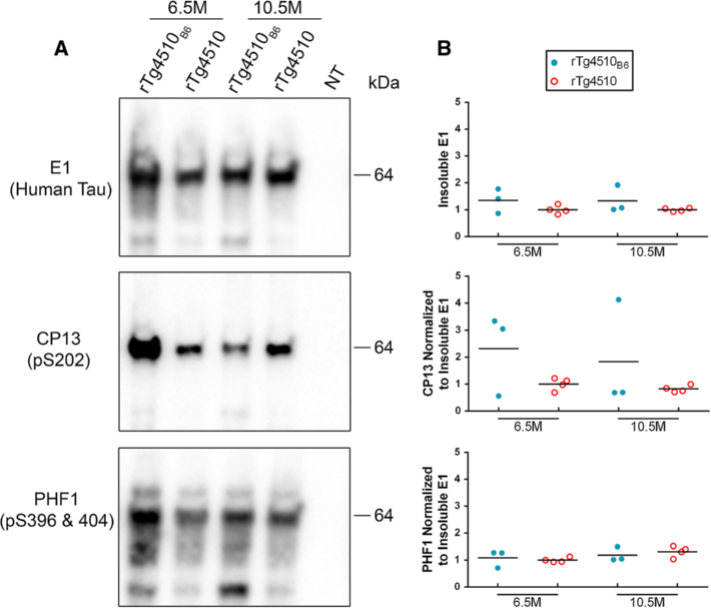
**Accumulation and phosphorylation of insoluble tau is not significantly changed in older rTg4510**_**B6 **_**mice. (A-B)** Western blot analyses of the sarkosyl-insoluble fractions of whole brain lysates from rTg4510_B6_ and rTg4510 mice at 6.5 and 10.5 months of age. **(A)** Representative western blots of insoluble human and phosphorylated tau with a NT littermate shown as a negative control. **(B)** Densitometric quantification of E1 (human tau), CP13 (pS202 tau) and PHF1 (pS396/404 tau). Phospho-tau is normalized to the amount of human tau aggregated in that fraction. Each dot represents an individual mouse with the mean indicated by the black line. n = 3-4 per cohort.

Having found that the onset of tauopathy is unchanged in rTg4510_B6_ mice compared to the original rTg4510 mice, we then sought to determine if the C57BL/6 background alters late stage tauopathy in mice at 6.5 and 10.5 months of age. Unlike young mice, old rTg4510_B6_ mice had similar levels of soluble human tau and increased levels of total soluble tau (human and mouse) as rTg4510 mice (p < 0.01) (Figure 6). Further, while there was not a significant difference in the phosphorylation of soluble tau detected with CP13, analysis with PHF1 revealed that old rTg4510_B6_ mice had twice as much soluble tau phosphorylated at S396/404 compared to old rTg4510 mice (p < 0.01) (Figure 6). By late stage tauopathy, rTg4510 mice were characterized primarily by a 64 kDa species of tau in the sarkosyl-insoluble brain fraction, regardless of strain background and the accumulation of human tau in the insoluble fraction was similar between rTg4510_B6_ and rTg4510 mice (Figure 7). The accumulation of human and mouse tau in the sarkosyl-insoluble fraction, as assessed with the Tau5 antibody, was also similar between rTg4510_B6_ and rTg4510 mice (data not shown). The 64 kDa insoluble tau band from rTg4510 mice on either background was similarly phosphorylated at epitopes associated with human tauopathy when normalized to the amount of human tau (Figure 7) or human and mouse tau (data not shown) in the sarkosyl-insoluble fraction. Results from the biochemical analysis of tau in older rTg4510_B6_ and rTg4510 mice are summarized in Table 1.

**Table 1:** **Two-way ANOVA of strain background and age effects on soluble and insoluble tau in 6.5 and 10.5 month-old rTg4510**_**B6 **_**mice compared to rTg4510 mice**

	**Strain Background**		**Age**		**Interaction**	
**Fraction stain**	**F (DFn, DFd)**	**P value**	**F (DFn, DFd)**	**P value**	**F (DFn, DFd)**	**P value**
**Soluble E1**^1^	F (1, 10) = 3.60	P = 0.09	F (1, 10) = 3.20	P = 0.10	F (1, 10) = 0.94	P = 0.36
**Soluble Tau5**^1^	F (1, 10) = 11.00	P < 0.01	F (1, 10) = 3.20	P = 0.10	F (1, 10) = 0.05	P = 0.82
**Soluble CP13**^1^	F (1, 10) = 2.40	P = 0.15	F (1, 10) = 0.06	P = 0.81	F (1, 10) = 0.25	P = 0.63
**Soluble PHF1**^1^	F (1, 10) = 20.00	P < 0.01	F (1, 10) = 4.80	P = 0.05	F (1, 10) = 0.57	P = 0.47
**Insoluble E1**	F (1, 10) = 3.90	P = 0.08	F (1, 10) = 0.002	P = 0.97	F (1, 10) = 0.002	P = 0.97
**Insoluble Tau5**	F (1, 10) = 2.70	P = 0.13	F (1, 10) = 1.20	P = 0.29	F (1, 10) = 0.50	P = 0.50
**Insoluble CP13**^2^	F (1, 10) = 3.60	P = 0.09	F (1, 10) = 0.29	P = 0.60	F (1, 10) = 0.06	P = 0.81
**Insoluble PHF1**^2^	F (1, 10) = 0.04	P = 0.85	F (1, 10) = 2.90	P = 0.12	F (1, 10) = 0.75	P = 0.41

^1^Normalized to GAPDH; ^2^Normalized to insoluble E1.

### rTg4510_B6_ mice have increased tau pathology compared to rTg4510 mice

**Figure 8:**
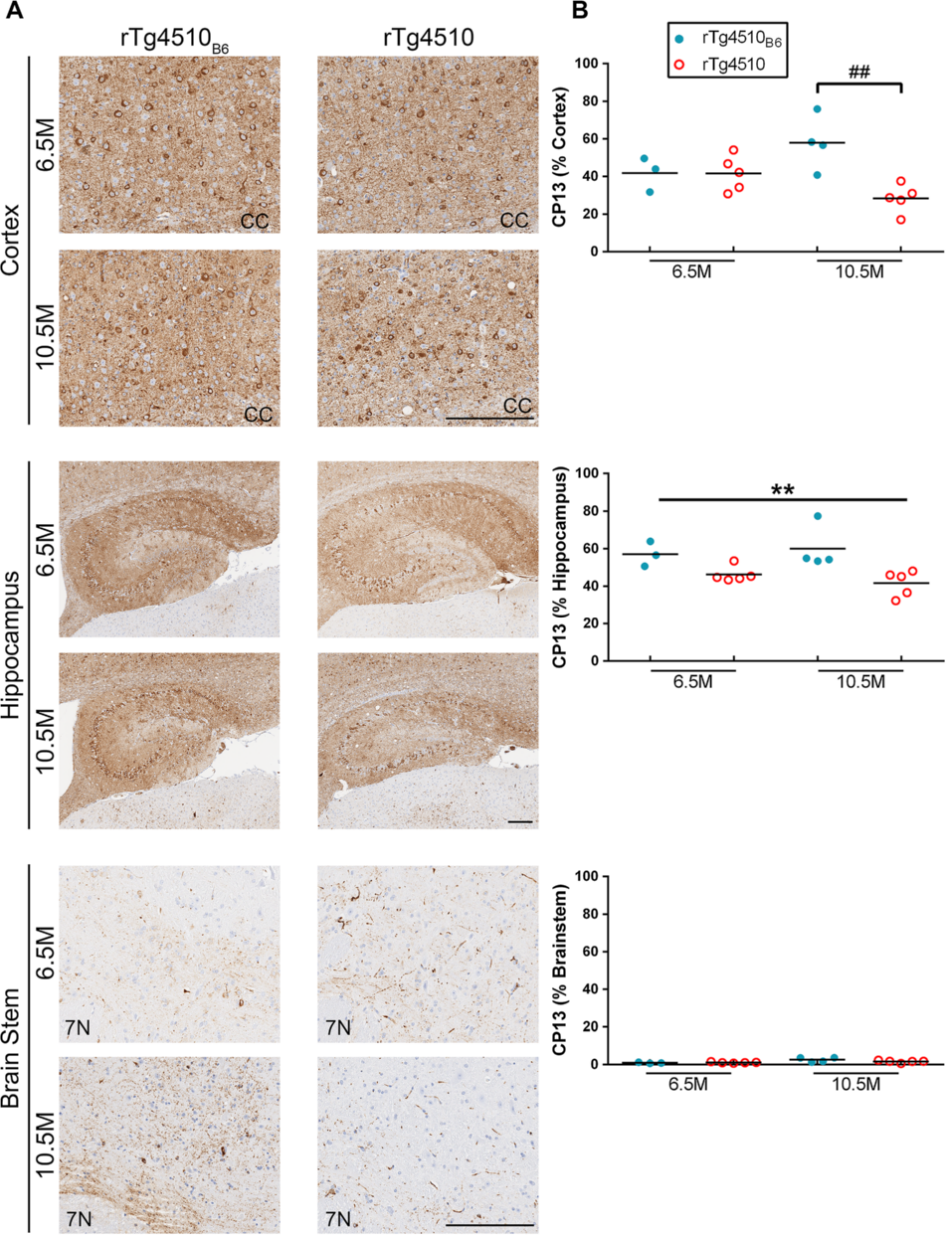
**Older rTg4510**_**B6 **_**mice have region-specific increases in CP13 compared to rTg4510 mice.** rTg4510_B6_ mice had increased CP13 (pS202 tau) staining in the cortex and hippocampus, but not the brainstem regions of the brain compared to rTg4510 mice at 10.5 months of age. **(A)** Representative images of cortical, hippocampal and brainstem regions of 6.5- and 10.5-month-old rTg4510_B6_ and rTg4510 mice stained with the CP13 antibody. **(B)** Quantitative analyses of the stained sections showed that the CP13 burden in the cortex of rTg4510_B6_ mice was significantly greater than the corresponding regions of rTg4510 mice at 10.5 months, but not 6.5 months of age, and increased in the hippocampus of rTg4510_B6_ at both age points. Each dot represents an individual mouse with the mean indicated by the black line, n = 3-5 per cohort. ***p < 0.001 [two-way ANOVA (strain x age): main effect of strain indicated]. ##p < 0.01 (*post hoc* Bonferonni’s multiple comparisons test). Scale bar represents 200 μm.

**Figure 9:**
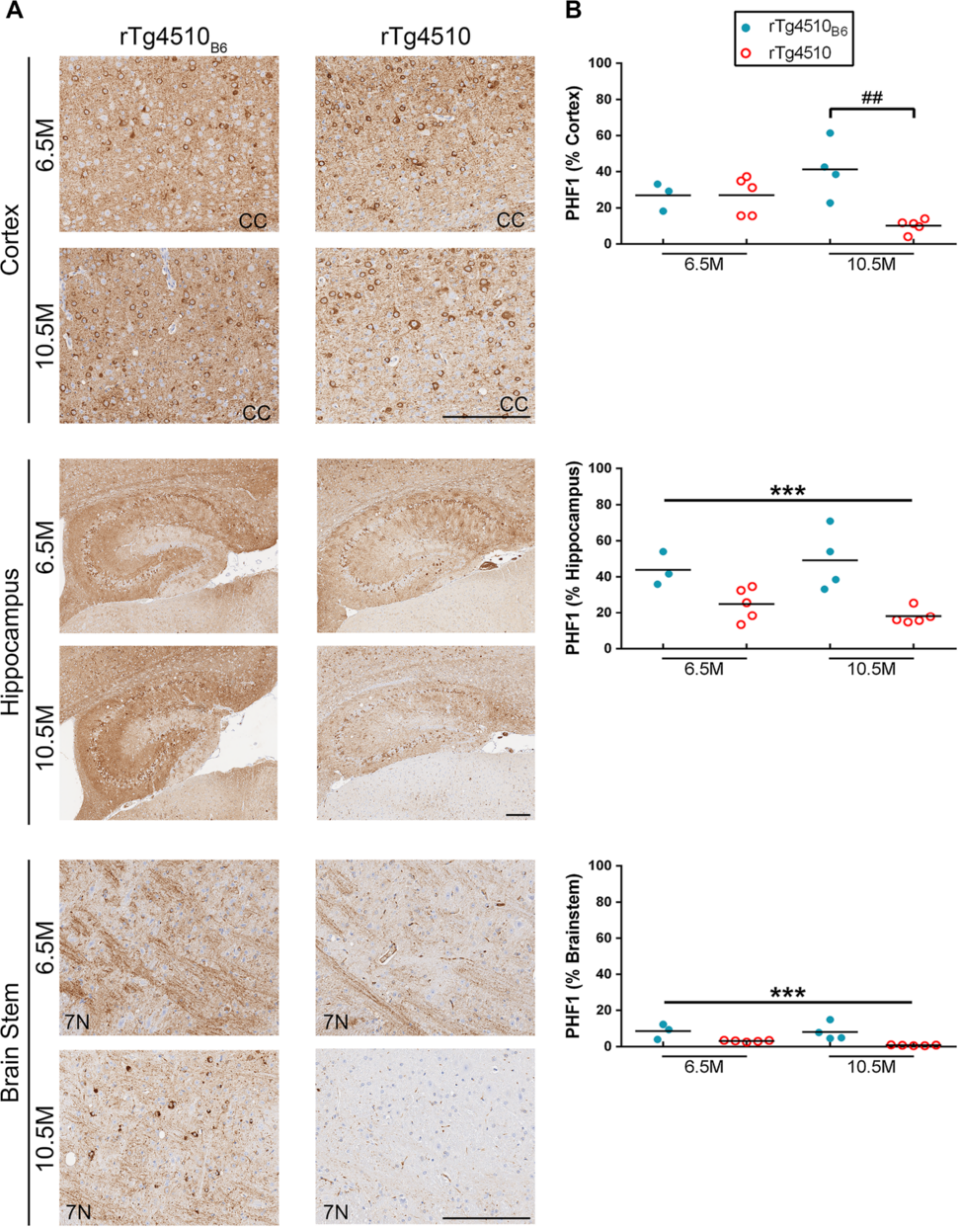
**Increased PHF1 tau staining in multiple brain regions of older rTg4510**_**B6 **_**mice.** Older rTg4510_B6_ mice had increased PHF1 (pS396/404 tau) staining compared to rTg4510 mice in the cortex, hippocampus and brainstem regions of the brain. **(A)** Representative images of cortical, hippocampal and brainstem regions from 6.5-and 10.5-month-old rTg4510_B6_ and rTg4510 mice stained with the PHF1 antibody. **(B)** Quantitative analyses of the stained sections showed that the PHF1 burden was significantly greater in the cortex of rTg4510_B6_ mice at 10.5 months and in the hippocampus and brainstem at both 6.5 and 10.5 months of age compared to the corresponding regions of rTg4510 mice. Each dot represents an individual mouse with the mean indicated by the black line, n = 3-5 per cohort. ***p < 0.001 [two-way ANOVA (strain x age): main effect of strain indicated]. ##p < 0.01 (*post hoc* Bonferonni’s multiple comparisons test). Scale bars represent 200 μm. CC = Corpus callosum; 7 N = 7^th^ Cranial nerve.

Immunohistochemical (IHC) staining with CP13 and PHF1 antibodies was used to compare tauopathy in rTg4510_B6_ and rTg4510 mice. Both CP13 and PHF1 detected striking amounts of tauopathy in the cortex and hippocampus of late stage rTg4510 mice on either background and tau pathology was found in both the cell body and the neuropil (Figures 8 and 9). The brainstem was minimally affected, reflecting the low levels of transgenic tau expression within this brain structure (Figures 8 and 9). Results from a two-way ANOVA (strain background x age) indicated that rTg4510_B6_ mice had increased cortical CP13-specific phospho-tau compared to rTg4510 mice [F (1, 13) = 8.60, p < 0.05], but there was an interaction of strain background and age [F (1, 13) = 8.40, p < 0.05] (Figure 8, Table 2). *Post hoc* analysis revealed that cortical CP13 burden was increased in rTg4510_B6_ mice at 10.5 months of age (p < 0.01), but not 6.5 months of age when compared to age-matched rTg4510 mice. Independent of age, CP13 burden was also significantly higher in the hippocampus of rTg4510_B6_ mice compared to rTg4510 mice [F (1, 13) = 15.00, p < 0.01] (Figure 8, Table 2). We also analyzed the brainstem of rTg4510_B6_ and rTg4510 mice and found that CP13 staining was increased with age but unaffected by the strain background of the mice (Figure 8, Table 2). Quantitation of regional PHF1 staining yielded similar results. Cortical PHF1 staining was increased in rTg4510_B6_ mice compared to rTg4510 mice [F (1, 13) = 9.20, p < 0.01], but there was an interaction of strain background and age [F (1, 13) = 9.40, p < 0.01] with PHF1 burden being significantly higher in rTg4510_B6_ mice over rTg4510 mice only at 10.5 months of age (p < 0.01) (Figure 9, Table 2). Additionally, PHF1 was significantly increased in the hippocampus of rTg4510_B6_ mice compared to rTg4510 mice at both 6.5 and 10.5 months of age [F (1, 13) = 23.00, p < 0.001] (Figure 9). Interestingly, there was also increased PHF1 immunoreactivity that was not seen with CP13 in the brainstem of rTg4510_B6_ mice when compared to rTg4510 mice on the original strain background [F (1, 13) = 21.00, p < 0.001] (Figure 9, Table 2). Overall, IHC analysis revealed age-dependent regional differences in phospho-tau pathology in mice crossed to a C57BL/6 background compared to mice on the original rTg4510 strain background.

**Table 2:** **Two-way ANOVA of strain background and age effects on phospho-tau burden in 6.5 and 10.5 month-old rTg4510**_**B6 **_**mice compared to rTg4510 mice**

	**Strain background**		**Age**		**Interaction**	
**Region stain**	**F(DFn, DFd)**	**P value**	**F(DFn, DFd)**	**P value**	**F(DFn, DFd)**	**P value**
**Cortex CP13**	F (1, 13) = 8.60	P < 0.05	F (1, 13) = 0.08	P = 0.78	F (1, 13) = 8.40	P < 0.05
**Cortex PHF1**	F (1, 13) = 9.20	P < 0.001	F (1, 13) = 0.05	P = 0.83	F (1, 13) = 9.40	P < 0.001
**Hipp CP13**	F (1, 13) = 15.00	P < 0.01	F (1, 13) = 0.05	P = 0.83	F (1, 13) = 1.00	P = 0.33
**Hipp PHF1**	F (1, 13) = 23.00	P < 0.001	F (1, 13) = 0.03	P = 0.88	F (1, 13) = 1.40	P = 0.26
**BS CP13**	F (1, 13) = 1.20	P = 0.29	F (1, 13) = 9.90	P < 0.01	F (1, 13) = 2.80	P = 0.12
**BS PHF1**	F (1, 13) = 21.00	P < 0.001	F (1, 13) = 1.10	P = 0.32	F (1, 13) = 0.43	P = 0.52

### Murine tau phosphorylation is influenced by strain background

**Figure 10:**
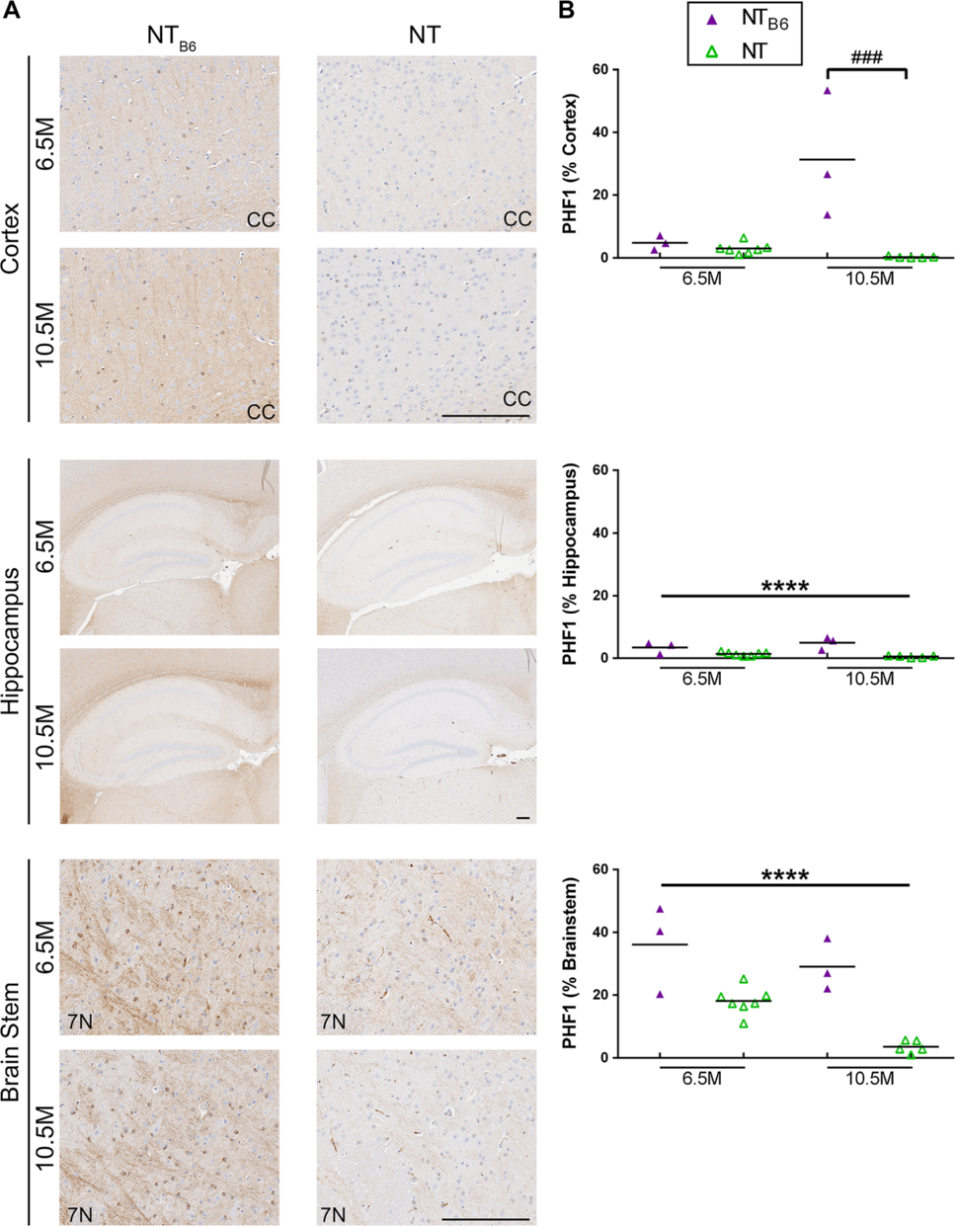
**Increased PHF1 staining in older NT**_**B6 **_**mice compared to NT mice.** NT mice on an F1 FVB/B6 background (NT_B6_) had increased PHF1 (pS396/404 tau) staining in the cortex, hippocampus and brainstem regions of the brain compared to NT mice on an F1 FVB/129 background (NT). **(A)** Representative images from the primary sensory cortex, hippocampal formation and brainstem of NT_B6_ and NT mice stained with PHF1. **(B)** Quantification of PHF1 burden in the cortex, hippocampus, and brainstem of 6.5- and 10.5-month-old NT_B6_ and NT mice. Cortical PHF1 staining was significantly increased in NT_B6_ mice compared to NT mice only at 10.5 months of age, while at 6.5 and 10.5 months of age PHF1 burden was increased in the hippocampal formation and brainstem. Each triangle represents an individual mouse with the mean indicated by the black line, n = 3-7 per cohort. ***p < 0.0001 [two-way ANOVA (strain x age): main effect of strain indicated]. ###p < 0.001 (*post hoc* Bonferonni’s multiple comparisons test). Scale bars represent 200 μm. CC = Corpus callosum; 7 N = 7^th^ Cranial nerve.

As human tau transgene expression in the brainstem of the original rTg4510 mice is very low, it was surprising to detect increased PHF1 staining in the brainstem of old rTg4510_B6_ mice. The PHF1 antibody recognizes both phosphorylated human and mouse tau; therefore, it was possible that that addition of C57BL/6 into the strain background could increase phosphorylation of endogenous mouse tau. To test this, we analyzed PHF1 staining in NT mice on both the F1 FVB/B6 (NT_B6_) and the F1 FVB/129 (NT) strain backgrounds. Similarly to rTg4510 mice, PHF1 staining was increased in the cortex of NT_B6_ mice compared to NT mice [F (1, 14) = 18.00, p < 0.001], but there was an interaction of strain background and age [F (1, 14) = 14.00, p < 0.01] with PHF1 burden being significantly higher in NT_B6_ mice over NT mice only at 10.5 months of age (p < 0.001) (Figure 10, Table 3). Additionally, PHF1 staining was significantly increased in the hippocampus and brainstem of NT_B6_ mice compared to NT mice at both time points and independent of an interaction with age [hippocampus: F (1, 14) = 33.00, p < 0.0001; brainstem: F (1, 14) = 40.00, p < 0.0001] (Figure 10, Table 3). Analysis with the CP13 antibody revealed that old NT_B6_ mice had significantly more CP13 staining in the cortex [F (1, 14) = 9.90, p < 0.01], but not in the hippocampus or brainstem, compared to old NT mice (data not shown, Table 3).

**Table 3:** **Two-way ANOVA of strain background and age effects on phospho-tau burden in 6.5 and 10.5 month-old NT**_**B6 **_**mice compared to NT mice**

	**Strain background**		**Age**		**Interaction**	
**Region stain**	**F(DFn, DFd)**	**P value**	**F(DFn, DFd)**	**P value**	**F(DFn, DFd)**	**P value**
**Cortex CP13**	F (1, 14) = 9.90	P < 0.01	F (1, 14) = 1.50	P = 0.24	F (1, 14) = 3.30	P = 0.09
**Cortex PHF1**	F (1, 14) = 18.00	P < 0.001	F (1, 14) = 9.30	P < 0.01	F (1, 14) = 14.00	P < 0.01
**Hipp CP13**	F (1, 14) = 1.30	P = 0.28	F (1, 14) = 0.04	P = 0.84	F (1, 14) = 0.01	P = 0.92
**Hipp PHF1**	F (1, 14) = 33.00	P < 0.0001	F (1, 14) = 0.32	P = 0.58	F (1, 14) = 4.50	P = 0.05
**BS CP13**	F (1, 14) = 0.03	P = 0.86	F (1, 14) = 0.28	P = 0.61	F (1, 14) = 3.20	P = 0.10
**BS PHF1**	F (1, 14) = 40.00	P < 0.0001	F (1, 14) = 9.80	P < 0.01	F (1, 14) = 1.20	P = 0.29

### Equivalent neurodegeneration in old rTg4510_B6_ and rTg4510 mice

**Figure 11:**
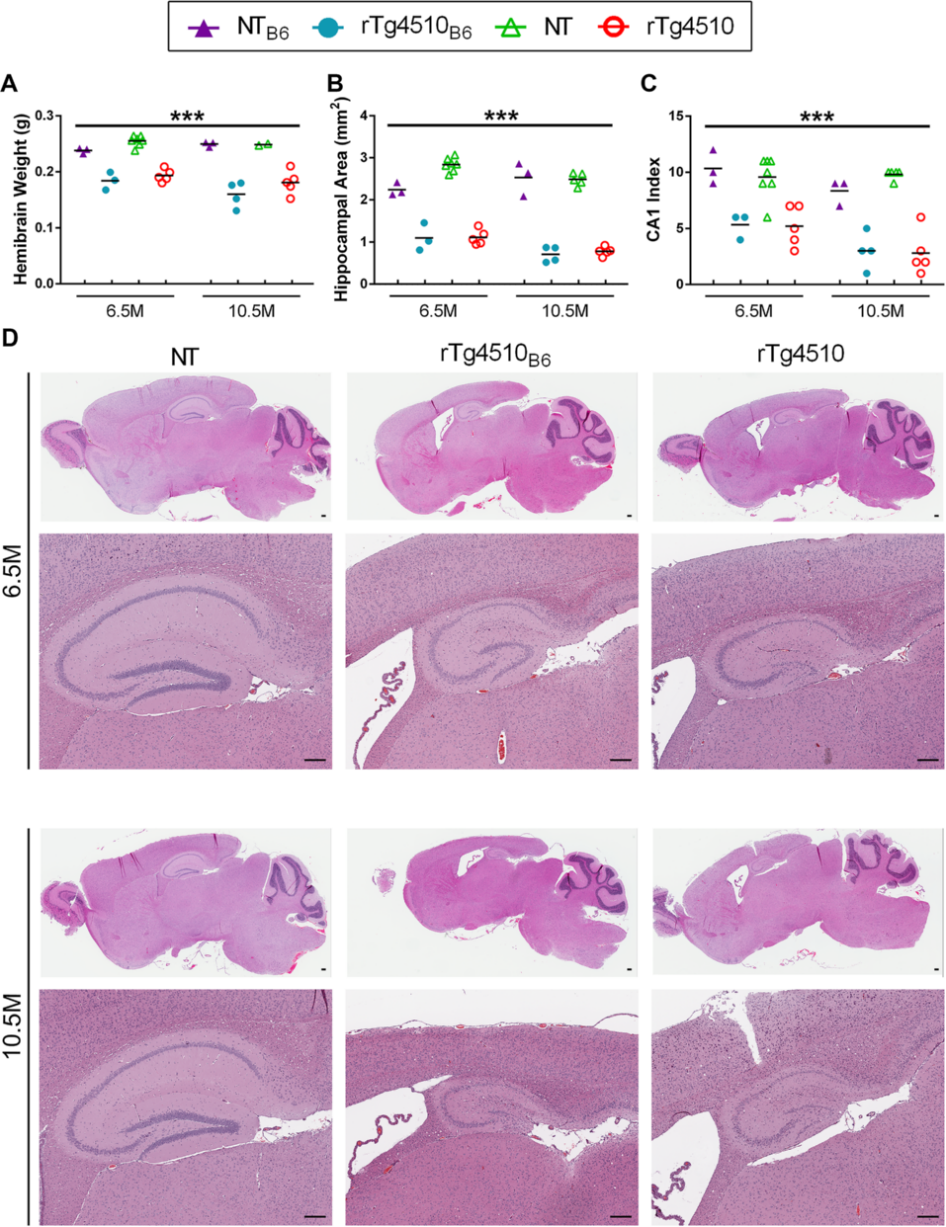
**Older rTg4510**_**B6 **_**mice have equivalent gross neurodegeneration.** Gross brain and hippocampal atrophy was equivalent in rTg4510_B6_ and rTg4510 mice at 6.5 and 10.5 months of age. **(A)** All mice on an F1 FVB/B6 background had smaller hemi-brains than all mice on an F1 FVB/129S background (p < 0.05). Independent of strain background, hemi-brain weights for rTg4510 mice were significantly decreased compared to NT mice. **(B)** Hippocampal atrophy, as assessed by decreased hippocampal area, was significantly increased in age-matched rTg4510 mice compared to NT littermates, with no difference between rTg4510 mice on different strain backgrounds. **(C)** rTg4510_B6_ and rTg4510 mice had similar CA1 index values that were significantly less than that of NT mice, indicating increased neuronal loss in the CA1 hippocampal sub-region. **(D)**  Overall, rTg4510 mice had gross atrophy of the forebrain and ventricle enlargement compared to NT mice that was indistinguishable between age-matched rTg4510 mice on different strain backgrounds. In rTg4510 mice, the hippocampal formation was visibly reduced in size with substantial thinning of the neuronal layers in the CA1 and dentate gyrus sub-regions. **(A-C)** Each dot represents an individual mouse with the mean indicated by the black line. Data was analyzed via multifactorial (age x genotype x strain) ANOVA. ***p < 0.001 (main effect of genotype indicated). n = 2-7 per cohort. Scale bars represent 200 μm.

A pathological hallmark of rTg4510 mice is age-dependent neurodegeneration of the forebrain regions that is detected by 5.5 months of age [2,14]. Consistent with this, 6.5-and 10.5-month-old rTg4510 mice on both strain backgrounds had significantly lower hemi-brain weights than NT mice [F (1, 24) = 158.72, p < 0.001] (Figure 11A). There was also a small, but significant decrease in the brain weight of all mice on an F1 FVB/B6 background compared to all mice on the original F1 FVB/129 background [F (1, 24) = 4.60, p < 0.05] that was independent of genotype. We then wanted to determine if there were differences in the size of regions specifically vulnerable to tau-related neurodegeneration in this model. Neuronal loss in forebrain regions of rTg4510 mice has been well characterized by work from Spires et al. that used stereological methods to demonstrate that the overall hippocampal formation has the greatest neuronal loss when compared to the cortical and striatal regions [18]. To assess hippocampal atrophy in rTg4510_B6_ mice, we measured the area of the hippocampal formation in matched sagittal sections of 6.5-and 10.5-month-old rTg4510 mice and NT littermates on both strain backgrounds. Three factor ANOVA (age x strain x genotype) revealed that the area of the hippocampi were significantly affected by age, strain background and genotype [age: F (1, 27) = 7.57, p < 0.01; strain: F (1, 27) = 5.07, p < 0.05; genotype: F (1, 27) = 501.97, p < 0.001], and that there was an interaction between all three factors [F (1, 27) = 5.93, p < 0.05]. *Post hoc* analysis revealed that both 6.5-and 10.5-month-old rTg4510 mice had significantly smaller hippocampal areas compared to NT mice (ps < 0.0001), with no difference in size between rTg4510 mice on the original strain background and rTg4510_B6_ mice (Figure 11B). Additionally, at 6.5 months of age the area of the hippocampi in NT_B6_ mice was significantly smaller than NT mice (p < 0.01), but this difference was abrogated by 10.5 months of age (Figure 11B). We also examined the CA1 layer, as this hippocampal sub-region has been reported to have an 82% reduction of neurons by 8.5 months of age in rTg4510 mice on the original strain background [18]. We calculated a CA1 index value for each mouse with a smaller index value indicating fewer CA1 neurons (see Methods). In agreement with previous data by Spires et al. [18], we found that rTg4510 mice had significantly smaller CA1 indices than NT mice [F (1, 27) = 96.54, p < 0.001], suggesting that rTg4510 mice have greater neuronal loss (Figure 11C). Additionally, there was no difference between strains, although the CA1 index value was found to significantly decreased with age [F (1, 27) = 8.67, p < 0.01], consistent with progressive neuronal loss previously reported in this model. Overall, histological analyses of rTg4510_B6_ brains at 6.5 and 10.5 months of age revealed substantial thinning of the neuronal cell layers of the CA1 and dentate gyrus hippocampal sub-regions, gross atrophy of the hippocampi and cortices and ventricle enlargement that was indistinguishable from age matched rTg4510 mice (Figure 11D).

## Discussion

rTg4510 mice that overexpress mutant human tau are widely used in both academia and industry to study modifiers of human tauopathy [19-33], yet rTg4510 mice on a different strain background have not been fully characterized. One of the most commonly used mouse strains is C57BL/6 and crossing to this background can affect a mouse model’s phenotype because of genetic and behavioral differences that exist amongst inbred mouse strains (reviewed in [34]). In this study we analyzed rTg4510 mice on a C57BL/6NTac background (rTg4510_B6_) near the onset of tauopathy at 2.5 months of age and during late stage tauopathy at 6.5 and 10.5 months of age. We found that at 2.5 months of age, rTg4510_B6_ mice had a small, but significant increase in soluble human tau levels and equivalent tau phosphorylation and aggregation as compared to age-matched rTg4510 mice on the original strain background. Further, cognition was equally impaired in young rTg4510_B6_ and rTg4510 mice as compared to NT littermates. During late stage tauopathy, rTg4510_B6_ mice displayed hyperphosphorylated insoluble tau and robust neurodegeneration that was equivalent to rTg4510 mice. Additionally, soluble phospho-tau detected with the PHF1 antibody was increased in rTg4510_B6_ mice compared to rTg4510 mice and 10.5-month-old rTg4510_B6_ mice had greater amounts of phospho-tau in the cortex and hippocampus when compared to age-matched rTg4510 mice. Finally, NT littermates of rTg4510_B6_ mice also had greater amounts of cortical and hippocampal phospho-tau at 10.5 months of age compared to NT littermates of rTg4510 mice. Interestingly, aged rTg4510 and NT mice containing a C57BL/6 background had greater variation in measured phospho-tau levels, therefore, a greater number of mice are needed to confirm that the findings of aged rTg4510_B6_ mice were not influenced by an outlier.

Contrary to our findings with rTg4510 mice, previous work with JNPL3 mice showed that backcrossing onto C57BL/6 strain background significantly delayed the onset of disease and altered the pathological presentation of the original model [3]. Although JNPL3 mice express the same P301L 0N4R tau as rTg4510 mice, there are several important differences between the models that may explain the disparate effects of the C57BL/6 strain on tauopathy in each model. The tau transgenic integration sites in JNPL3 and rTg4510 mice have not been published, but it is likely that the incorporation of the human tau cDNA into the murine genome occurred at different locations for each model. Based on the integration site of the transgenes and the endogenous activity of that chromosomal region, strain background could differentially affect promoter activity in each model [35]. Moreover, the transgenic tau expression in JNPL3 mice is controlled by the mouse prion promoter, resulting in human mutant tau that predominantly affects neurons in the spinal cord and hindbrain. In contrast, tau expression in rTg4510 mice is ultimately controlled by the CaMKIIα promoter, which results in high tau expression in neurons in the forebrain region. The promoter affects which populations of neurons are expressing the transgene and different neuronal populations may be differently affected in each strain background. Additionally, the C57BL/6 sub-strain utilized in each study could also contribute to the difference. In this study, we crossed tau-expressing mice to a C57BL/6NTac (B6/NTac) sub-strain while Bolmont et al. crossed tau-expressing mice to a C57BL/6 J (B6/J) sub-strain. The B6/NTac sub-strain from Taconic was originally derived from the B6/J strain from Jackson laboratory, but over time genetic and phenotypic differences have developed between the two sub-strains [36-38]. Although we detected significant, but relatively modest changes in the tauopathy of rTg4510 mice on a B6/NTac background, it is possible that modifiers of tauopathy exist on the B6/J strain. Finally, the C57BL/6 strain zygosity has been shown to affect transgenic phenotype [4,5] and the Bolmont et al. study used congenic C57BL/6 mice while our studies used hybrid F1 C57BL/6 mice. If a recessive gene were responsible for altering tauopathy in JNPL3, such effects would not be detected in F1 rTg4510_B6_ mice.

In addition to the concerns detailed above, differences in transgenic protein levels could confound comparisons of the same model on different strain backgrounds (i.e. rTg4510_B6_ vs. rTg4510). By comparing tau protein levels between rTg4510_B6_ and rTg4510 mice prior to substantial neuronal loss, we found that rTg4510_B6_ mice have a small, but significant, increase in soluble human tau. Interestingly, this difference in soluble human tau levels was not detected using an antibody specific for both human and mouse tau, which may reflect a difference in endogenous tau levels between the two strain backgrounds. It should be noted though, that it is currently unclear if the Tau5 antibody has equal affinity for human and mouse tau, confounding such an interpretation. Increased soluble human tau in young rTg4510_B6_ mice could result from decreased tau degradation or from increased tau transgene expression. Because the tau responder line utilized in the rTg4510_B6_ and rTg4510 lines were identical, it is unlikely that the difference in transgenic tau expression arose from the tau responder line, per se. Since human tau expression in the rTg4510 model is dependent on both the tau and the tTA transgenes, elevated levels of tTA expression could underlie the increased human tau levels. The CaMKIIα-tTA mice used in rTg4510 and rTg4510_B6_ mice originated from the same line and laboratory [8] and they were subsequently backcrossed to different mouse strains by the two different laboratories from which we acquired the mice. It is possible that change of transgene copy number may have occurred between the two tTA sub-lines, and this could influence our findings. This can occur if there is inherent instability of the transgene locus [39]. We attempted to use commercially available antibodies raised against tTA to determine if tTA levels were changed between rTg4510_B6_ and rTg4510 mice, but like others, we were unsuccessful in detecting tTA protein using these antibodies [5,9,10]. We, therefore, were unable to determine if altered tTA expression underlies the modestly increased tau levels in young rTg4510_B6_ mice.

Despite increased human tau expression in 2.5-month-old rTg4510_B6_ mice, we did not detect differences in tau phosphorylation or aggregation at this age. Furthermore, rTg4510_B6_ and rTg4510 mice were similarly cognitively impaired. The hidden platform version of MWM revealed that cognition was equivalently impaired in 2.5-month-old rTg4510 mice across strain backgrounds. Interestingly, performance in the cued version of the MWM task showed a difference in the swim path to the visible platform between rTg4510 and NT mice across all days. Closer analysis of individual training days revealed that although rTg4510 mice on both backgrounds took significantly longer paths to reach the platform on days 1 and 2, by day 3 they performed comparably to NT littermates. This suggests that rTg4510 mice took a longer time to learn the cue, but once learned, were able to perform comparably to NT mice so that the deficits observed in rTg4510 mice on both backgrounds strains can be accredited to cognitive deficits rather than an inability to complete the task. Overall, although behavioral differences have been reported between C57BL/6 and 129S6 inbred strains [40,41], we found no differences in sensorimotor function or spatial dependent learning in mice of the same genotype and on an F1 FVB/B6 versus an F1 FVB/129 strain background.

Interestingly, aged (6.5-and 10.5-month-old) rTg4510_B6_ mice did not have significantly different human tau levels, as evaluated by soluble human tau. Total soluble tau levels, though, were significantly increased in aged rTg4510_B6_ mice as compared to rTg4510 mice when an antibody specific to both human and mouse tau was used. Assessment of tau protein production in old mice, however, is complicated by significant neurodegeneration as those neurons expressing human tau are likely the neurons that have died or will die. Given this, the modest but significantly elevated levels of human tau expression in the 2.5-month-old rTg4510_B6_ mice compared to rTg4510 are likely to be more accurate.

In old mice, the biochemical analysis of tau revealed increased phospho-tau in the soluble brain fraction of rTg4510_B6_ mice compared to rTg4510 mice using the PHF1 antibody. Additionally, IHC analysis revealed that PHF1 staining was increased in both the forebrain and hindbrain of rTg4510_B6_ mice, while CP13 burden was increased in forebrain regions (cortex and hippocampus), but not in the brainstem of old rTg4510_B6_ mice compared to old rTg4510 mice. Furthermore, NT_B6_ mice also had increased PHF1 and CP13 staining compared to NT mice that was spatiotemporally parallel to the increased PHF1 and CP13 staining in rTg4510_B6_ mice versus rTg4510 mice. Importantly, analysis of 64 kDa tau in the biochemically abnormal, sarkosyl-insoluble fraction and gross assessments of neurodegeneration through hemi-brain weights, hippocampal areas, and CA1 neuronal thickness, indicated no differences between rTg4510 mice on different strain backgrounds. Taken together, these results suggest that the differences we observed in soluble phosphorylated tau levels and phospho-tau burden of rTg4510_B6_ mice could be attributed to increased phosphorylation of endogenous mouse tau rather than transgenic human tau. Regardless, the enhanced murine tau phosphorylation does not appear to alter to ultimate neurodegenerative phenotype observed in rTg4510_B6_ mice. Interestingly, it has been reported that *Dab1* deficient mice on a C57BL/6 strain background have increased murine tau phosphorylation compared to *Dab1* deficient mice on a BALB/cByJ strain background [42]. Further, the increased murine phospho-tau has been correlated with higher expression of a novel modifier of tau phosphorylation, *Stk25*, in C57BL/6 mice [43]. In *Dab1* deficient mice on a C57BL/6 background, increased phosphorylation of mouse tau was not associated with overt neurodegeneration. These findings highlight a critical consideration when studying phospho-tau alterations in mice that contain both mouse and human tau.

## Conclusions

Here we report that introduction of the C57BL/6 strain into the rTg4510 mouse background minimally alters the presentation of tau pathology that was originally reported in rTg4510 on the F1 FVB/129 background. At 2.5 months of age, rTg4510_B6_ mice had minor increases in human tau expression compared to rTg4510 mice, but cognitive decline and phosphorylation of tau was equivalent in rTg4510 mice on both strain backgrounds. Late stage tauopathy in rTg4510_B6_ mice was robust with equivalent insoluble tau aggregation and gross neurodegeneration to rTg4510 mice. Additionally, we observed minor, but significant, increases in tau phosphorylation in both older rTg4510_B6_ mice as compared to rTg4510 mice, and in older NT_B6_ mice as compared to NT mice. Overall, these studies provide evidence that rTg4510 mice can be crossed to a B6/NTac background without loss or delay of the original phenotype.

## Methods

### Mice

The generation of the rTg4510 mouse model that uses a system of responder and activator transgenes to express human mutant (P301L) tau has been previously described [2]. Briefly, mice carrying a responder gene with human tau_P301L_ cDNA downstream of a tetracycline response element (TRE) were maintained on an FVB/N background while mice carrying an activator gene with the tetracycline transactivator (tTA) downstream of a calcium/calmodulin kinase IIα (CaMKIIα) promoter were maintained on a 129S6 background (tTA_129S_). TRE-tau responder mice are crossed with tTA_129S_ activator mice to produce rTg4510 mice on an F1 FVB/129S background with human mutant tau expression focused within forebrain. tTA_129S_ mice were obtained by crossing offspring carrying the CaMKIIα-tTA transgene [8] with 129S6/SvEv mice purchased from Taconic [2] (Figure 1). The original tTA_129S_ mice were acquired from George Carlson. Concurrently, tTA mice were maintained on a B6 background (tTA_B6_) by crossing the CaMKIIα-tTA mice [8] to C57BL/6NTac mice (Taconic) for more than ten generations [44]. The original tTA_B6_ mice were acquired from Li-Huei Tsai. To test the effects of the B6 strain background on the progression of tauopathy in the rTg4510 mouse model, we crossed TRE-tau responder mice to tTA_B6_ activator mice to produce rTg4510_B6_ mice on an F1 FVB/B6 genetic background (Figure 1). The initial characterization of rTg4510 indicated that single transgenic (tau only and tTA only) and non-transgenic (NT) littermates have similar performances in Morris water maze, so to maximize the number of rTg4510 mice that could be run within the same test, only NT mice were used as controls. Cohorts included 2.5-, 6.5-and 10.5-month-old rTg4510, rTg4510_B6_ and NT littermates of both sexes (see Table 4 for details). Grubb’s analysis of the western blot of sarkosyl-insoluble tau (see protocol below) identified one 2.5-month-old male rTg4510 mouse as an outlier due to extremely high tau aggregation. All western blot data from this mouse was excluded from the final results as this may have been a tissue preparation error.

**Table 4:** Age, number and sex of mice used in each experimental procedure

**Age**	**Analysis**	**rTg4510**_**B6**_	**NT**_**B6**_	**rTg4510**	**NT**
**2.5 Months**	MWM	5 F	3 F, 2 M	2 F, 3 M	2 F, 3 M
	WB	6 F, 4 M	1 F, 1 M	5 F, 4 M	1 F, 1 M
**6.5 Months**	WB	2 F, 1 M	-	4 F	-
	IHC	2 F, 1 M	3 M	5 F	5 F, 2 M
**10.5 Months**	WB	1 F, 2 M	2 M	2 F, 2 M	1 F, 1 M
	IHC	1 F, 3 M	4 M	2 F, 3 M	3 F, 2 M

Key: MWM = Morris water maze; WB = Western blot; IHC = Immunohistochemistry; NT = Non-transgenic; F = Female; M = Male.

Mice were housed and treated in accordance with the NIH Guide for the Care and Use of Laboratory Animals. All animal procedures were approved and conducted in accordance with the Mayo Clinic Institutional Animal Care and Use committee and the University of Florida Animal Care and Use committee. Mice were maintained in a pathogen-free facility on a 12 hour light/dark cycle with water and food provided *ad libitum*.

### Morris water maze

rTg4510, rTg4510_B6_ and NT littermates of both sexes underwent Morris water maze (MWM) testing at 2.5 months of age. Mice were handled for 6 days prior to the initiation of behavioral training. MWM testing was performed in a 60 cm high and 1.5 m diameter circular pool filled with water maintained between 24 and 26°C and that was made opaque using non-toxic paint. The swim path of each mouse was recorded by a video camera suspended 2.5 m over the center of the pool and connected to the video tracking system (HVS Image Advanced Tracker VP2000, HVS Image, Buckingham, UK). Curtains surrounding a portion of the pool were used to block the view of the computer. Both the curtains and walls of the room contained dark, geometric shapes to be used as visual cues.

MWM testing occurred over 6 consecutive days with task acquisition training on days 1 through 5 and with probe trials to evaluate spatial memory on day 3, prior to the onset of the training session, and on day 6. For training sessions, a 15 cm diameter platform was submerged 1 cm under the surface of the water in the middle of the target quadrant. The hidden escape platform was located in the same target quadrant for each session for each mouse. The mouse was transported to the pool in a holding container and released facing the wall of the pool from each of the cardinal directions in a semi-random fashion. Once released, the mouse had 60 seconds to locate the hidden platform. If a mouse failed to find the platform, then it was gently guided to the platform and each mouse was allowed to remain on the platform for 10 seconds. Each mouse received 4 training trials per day. During the probe trials on days 3 and 6, the hidden platform was removed from the pool and each animal searched for the hidden platform for 60 seconds.

Prior to the start of the MWM, the 2.5-month-old cohort underwent a visible cued test to evaluate sensorimotor function. A curtain surrounded the MWM tank so that spatial cues could not be referenced and a platform 0.5 cm above the water that contained a visual cue (a tall black rod) was placed in the tank. Both the quadrant location of the visible cued platform and the mouse release points were changed semi randomly for each trial to prevent habituation to a particular quadrant. Mice were given 90 seconds to reach the platform before being gently guided to the platform where the mice remained for 10 seconds. Each mouse was tested 4 times per day for 3 days.

### Tissue harvest and preparation

Mice were euthanized by cervical dislocation and the brains were rapidly removed and cut down the midline. The left hemisphere was drop fixed in 10% formalin for immunohistochemical analysis and the right hemisphere was frozen on dry ice and then stored at-80°C. For tau biochemical analysis, sarkosyl fractionation was performed on the frozen brains as previously described [2]. Specifically, each whole hemisphere was homogenized in 10 volumes of homogenate buffer [50 mM Tris–HCl, 274 mM NaCl, 5 mM KCl, 1% protease inhibitor mixture (Sigma), 1% phosphatase inhibitor cocktails I and II (Sigma), and 1 mM phenylmethylsulfonyl fluoride (PMSF), (pH 8.0)] and 200 μl of homogenate was then centrifuged at 150,000 x g for 15 minutes at 4°C. The supernatant, which contains the soluble tau species, was collected and the protein concentration determined using a standard BCA protein assay (Pierce). Pellets were further homogenized in 3 volumes (600 μl) of Buffer B [10 mM Tris–HCl (pH 7.4), 0.8 M NaCl, 10% Sucrose, 1 mM EGTA and 1 mM PMSF] and centrifuged for 15 minutes at 150,000 x g at 4°C. The supernatants were incubated with 1% sarkosyl (Sigma) for one hour at 37°C and then centrifuged at 150,000 x g for 30 minutes at 4°C. The sarkosyl-insoluble pellet was re-suspended in 20 μl TE buffer [10 mM Tris–HCl (pH 8.0), 1 mM EDTA] to obtain the biochemical equivalent of neurofibrillary tangles.

### Antibodies

Tau antibodies used included the polyclonal antibody E1 (specific for amino acids 19-33 of human tau) [45] and the monoclonal antibody Tau5 (provided by Dr. Lester I. Binder, Northwestern University Medical School) for western blot analysis and the monoclonal tau antibodies CP13 (specific for pS202 tau) and PHF1 (specific for pS396/404 tau) (provided by Dr. Peter Davies, The Einstein Institute for Medical Research, Manhasset, NY) for both western blot and immunohistochemical analysis. Anti-GAPDH (Biodesign) was used as a loading control for western blot analysis.

### Western blotting

For tau protein analysis, 1-5 μg of the soluble fraction and 3 μl (6.5-and 10.5-month-old mice) or 4.5 μl (2.5-month-old mice) of the sarkosyl-insoluble fraction were loaded in each well. Brain lysates were diluted in Novex Tris-glycine SDS-sample buffer (Invitrogen) with β-mercaptoethanol and heat denatured at 95°C for 5 minutes before being loaded onto 26-well 10% Tris-glycine gels (Invitrogen) and separated by SDS-PAGE. Protein was transferred in CAPS transfer buffer (Sigma) to PVDF membranes. Membranes were then blocked in Tris buffered saline plus 0.1% TritonX-100 (TBS-T) with 5% non-fat milk and incubated overnight with antibody diluted in TBS-T/5% milk. Membranes were washed in TBS-T, incubated with peroxidase-conjugated goat anti-mouse HRP or goat anti-rabbit HRP secondary antibodies (Jackson ImmunoResearch) for 1 hour at room temperature and washed again in TBS-T. Membranes were developed using Western Lightning Plus (Perkin Elmer) and imaged using a FluorChem E System (ProteinSimple). The relative levels of immunoreactivity were determined by densitometry using the software AlphaView SA (ProteinSimple). Relative soluble tau and phospho-tau levels were quantified by normalizing protein levels to GAPDH levels. The relative levels of phospho-tau in the sarkosyl-insoluble fraction were determined by normalizing CP13 and PHF1 levels with E1 levels and Tau5 levels (data not shown) for each mouse to correct for the amount of tau aggregated in the sarkosyl-insoluble fraction of that animal. Tau and phospho-tau levels are presented relative to 6.5-month-old rTg4510 mice for the older cohorts (6.5 and 10.5 months of age) and relative to 2.5-month-old rTg4510 mice for the young cohort (2.5 months of age).

### Immunohistochemical analysis

Fixed mouse brains were paraffin embedded and cut into 5 μm sagittal sections. Hematoxylin and eosin (H&E) staining was performed on at least two brain sections from each mouse to align all brains to approximately 1.3 mm lateral to the midline using a mouse brain atlas [46]. Stained slides were digitally scanned using a Scanscope XT scanner. Hippocampal area measurements were performed on a sagittal section stained with H&E obtained for each mouse. The hippocampal formation was then outlined using ImageScope version 10 software (Aperio, Vista, CA) and the resultant area value for each animal was used to generate group means. The same section used for hippocampal area measurements per animal was also used to calculate the CA1 index. Three lines were pseudo-randomly drawn perpendicular to the main axis of the CA1 cell layer and the number of intact neuronal cell bodies touching each line was blindly counted. Each of the counts was summed to obtain a “CA1 index” value per animal.

Standard immunohistochemical procedures were used to immunostain tissues with PHF1 and CP13 antibodies and counterstain with hematoxylin using the Dako Universal Autostainer with DAKO Envision + HRP system (Dako). Stained slides were digitally scanned using a Scanscope XT scanner and were analyzed using ImageScope software. Positive pixel count algorithms were created to measure the percent positivity of the secondary antibody, specifically chromogen DAB, in a selected region. Distinct algorithms were used for burden analysis of each primary antibody in either rTg4510 or NT littermates. The brain regions analyzed included the cortex and the hippocampal formation, which expressed high levels of human tau, and the brain stem, which has very low human tau expression in rTg4510 mice.

### Statistical analysis

Results for MWM training are displayed as the group mean ± SEM and were analyzed using multifactorial repeated measures (RM) ANOVA, with strain background and genotype as between subject factors and training days as within subject factors. When appropriate, *post hoc* comparisons were performed. The probe trial for MWM was analyzed using two-way ANOVA (genotype x strain) with *post hoc* Bonferroni’s multiple comparisons test. Western blot results from 2.5-month-old mice were analyzed with an unpaired, two tailed Student’s t-test. Assessments of gross neuropathology in 6.5- and 10.5-month-old rTg4510 and rTg4510_B6_ mice and NT_B6_ littermates were analyzed using one-way ANOVA with *post hoc* analysis with Bonferroni’s multiple comparison tests. Western blot and IHC data from 6.5- and 10.5-month-old rTg4510 and rTg4510_B6_ mice were analyzed using two-way ANOVA with strain background and age as independent variables and *post hoc* analysis with Bonferroni’s multiple comparison test. Grubb’s analysis was used to identify outliers in the western blot analysis of sarkosyl-insoluble tau. Analyses were performed using GraphPad Prism version 6.00 software (GraphPad Software) and SPSS version 20.0 (IBM). In all cases, p < 0.05 was considered to be statistically significant.

## Abbreviations

TRE: Tetracycline response element; CaMKIIα: Calcium/calmodulin kinase IIα; B6: C57BL/6 mouse strain; NT: Non-transgenic mice on an FVB/N x 129S6 strain background; NTB6: Non-transgenic mice on an FVB/N x C57BL/6 N strain background; tTA: Tetracycline transactivator; tTA129S: Mice with a tTA transgene on a 129S6 strain background; tTAB6: Mice with a tTA transgene on a C57BL/6 N strain background; rTg4510: Mice with tau responder and tTA transgenes on an FVB/N x 129S6 strain background; rTg4510B6: Mice with tau responder and tTA transgenes on an FVB/N x C57BL/6 N strain background; MWM: Morris water maze; IHC: Immunohistochemical.

## Competing interests

JL holds IP and has received royalties in excess of $10,000 NIH threshold for significant conflict of interest in the past year from the rTg4510 mouse model.

## Authors’ contributions

RMB, carried out animal breeding and harvests, tissue processing, immunohistochemical studies, data analysis, manuscript write up and aided in conceptualization of study. JH, performed animal behavioral tests, western blotting, data analysis and contributed to manuscript write up. JK, carried out animal breeding and harvesting. NS, contributed to western blotting. DWD, contributed to the generation of stained mouse brain sections for immunohistochemical analysis and manuscript editing. JL, conceived study and interpreted data, contributed research animals and performed manuscript editing. All authors read and approved the final manuscript.
